# The efficacy of Carbamylglutamate impacts the nutritional management of patients with N-Acetylglutamate synthase deficiency

**DOI:** 10.1186/s13023-024-03167-0

**Published:** 2024-04-18

**Authors:** Rani H. Singh, Marie-Hélène Bourdages, Angela Kurtz, Erin MacLoed, Chelsea Norman, Suzanne Ratko, Sandra C. van Calcar, Aileen Kenneson

**Affiliations:** 1 Emory University School of Medicine, 101 Woodruff Circle, 7th Floor Suite 7130, 30322 Atlanta, GA USA; 2 Children’s University Hospital of Quebec, Quebec, QC Canada; 3 Mount Sinai, New York, NY USA; 4 Children’s National Medical Center, Washington, DC USA; 5 University of Utah, Salt Lake City, UT USA; 6 London Health Sciences Centre, London, ON Canada; 7 Oregon Health & Science University, Portland, OR USA

**Keywords:** N-acetylglutamate synthase, NAGS deficiency, Carbamylglutamate, Urea cycle disorder, Hyperammonemia

## Abstract

**Background:**

The autosomal recessive disorder N-acetylglutamate synthase (NAGS) deficiency is the rarest defect of the urea cycle, with an incidence of less than one in 2,000,000 live births. Hyperammonemic crises can be avoided in individuals with NAGS deficiency by the administration of carbamylglutamate (also known as carglumic acid), which activates carbamoyl phosphatase synthetase 1 (CPS1). The aim of this case series was to introduce additional cases of NAGS deficiency to the literature as well as to assess the role of nutrition management in conjunction with carbamylglutamate therapy across new and existing cases.

**Methods:**

We conducted retrospective chart reviews of seven cases of NAGS deficiency in the US and Canada, focusing on presentation, diagnosis, medication management, nutrition management, and outcomes.

**Results:**

Five new and two previously published cases were included. Presenting symptoms were consistent with previous reports. Diagnostic confirmation via molecular testing varied in protocol across cases, with consecutive single gene tests leading to long delays in diagnosis in some cases. All patients responded well to carbamylglutamate therapy, as indicated by normalization of plasma ammonia and citrulline, as well as urine orotic acid in patients with abnormal levels at baseline. Although protein restriction was not prescribed in any cases after carbamylglutamate initiation, two patients continued to self-restrict protein intake. One patient experienced two episodes of hyperammonemia that resulted in poor long-term outcomes. Both episodes occurred after a disruption in access to carbamylglutamate, once due to insurance prior authorization requirements and language barriers and once due to seizure activity limiting the family’s ability to administer carbamylglutamate.

**Conclusions:**

Follow-up of patients with NAGS deficiency should include plans for illness and for disruption of carbamylglutamate access, including nutrition management strategies such as protein restriction. Carbamylglutamate can help patients with NAGS deficiency to liberalize their diets, but the maximum safe level of protein intake to prevent hyperammonemia is not yet known. Patients using this medication should still monitor their diet closely and be prepared for any disruptions in medication access, which might require immediate dietary adjustments or medical intervention to prevent hyperammonemia.

## Introduction

Detoxification of ammonia occurs via the urea cycle and is dependent on the function of six enzymes and two mitochondrial transporters. Deficiency in one of these enzymes, N-acetylglutamate synthase (NAGS), inherited as an autosomal recessive disorder (17q21.31), is the rarest defect of the urea cycle, with an incidence of less than one in 2,000,000 live births [1]. NAGS is a mitochondrial enzyme that catalyzes the formation of N-acetylglutamate (NAG) [2], an essential activator of carbamoyl phosphate synthetase 1 (CPS1), the first enzyme of the urea cycle in mammals [3, 4]. Thus, NAGS deficiency results in dysregulation of ammonia detoxification [5, 6], which can lead to an array of symptoms with variable severity based on mutation status [7], including encephalopathy, coma, and death. Given the rarity of the disorder, it is unusual to have information on multiple cases, though several case reports have been published describing individual patient experiences [8].

The majority of patients with NAGS deficiency are diagnosed in infancy. In a review of 98 published cases of NAGS deficiency, 58% presented before the age of one month [8]. Presentation directly after birth includes poor feeding or feeding intolerance, vomiting, lethargy, hypertonia and/or hypotonia, seizures, and tachypnea. Common presenting symptoms in later onset cases include vomiting, confusion or disorientation, ataxia, lethargy, decreased level of consciousness, seizures, hypotonia, and in some cases, avoidance of high-protein foods [8]. Because it is possible to present apparently normal ammonia detoxifying function with partial NAGS deficiency, some cases can be difficult to diagnose until a hyperammonemic crisis occurs [1].

Treatment of acute hyperammonemia generally includes the use of nitrogen scavengers such as benzoate and phenylacetate, as well as the administration of carbamylglutamate [9]. Also known as carglumic acid, carbamylglutamate is a synthetic form of NAG that activates the CPS1 enzyme [10]. Once ammonia levels are stabilized and NAGS deficiency is diagnosed, treatment focuses on avoiding a future crisis event. Historically, this was done via limiting protein intake and avoiding catabolic stress [11]. However, the addition of long-term carbamylglutamate use has proven effective in allowing a more liberalized protein intake [5, 12–14].

Diagnosing and treating NAGS deficiency has been complicated by the variety of driver mutations, with over 50 mutations reported, and their associated symptoms and disease severity [8, 15]. Currently, there is a lack of consolidated and up-to-date literature that comprehensively covers cases of NAGS deficiency, particularly in understanding the area of nutritional interventions. Therefore, published descriptions of patients with NAGS deficiency, including presentation, recurring symptoms, and treatment, is essential to better understanding this condition. In this case series, we consolidate clinical information among seven treated patients with NAGS deficiency to provide insight into diagnosis and treatment, with a focus on medication and nutrition management, to contribute to the data supporting best practices.

## Methods

This case series includes seven cases of NAGS deficiency treated in the United States (US) and Canada. Cases were identified by querying members of the Emory University Genetic Nutrition Online Metabolic listserv, which includes all Genetic Metabolic Dietitians International group members, as well as other practitioners who treat patients with inherited metabolic disorders (IMDs) or who have an interest in the nutrition management of patients with IMDs. We initiated discussions with an in-person meeting of registered dietitians who reported that they had managed a patient with NAGS deficiency. The meeting consisted of a didactic session followed by case presentations and group discussions. At this meeting and during subsequent follow-up telephone conferences, we developed a standardized data collection form to record retrospective information from medical records on clinical presentation, diagnostic procedures, medical interventions (including carbamylglutamate, citrulline, and arginine doses), nutrition interventions, and results of biochemical tests at various stages during diagnosis and treatment.

Data were collected and pooled using the online REDCap database housed at Emory University. REDCap is a secure, HIPAA-compliant, web-based application designed to support data capture for research studies, providing: (1) an intuitive interface for validated data entry; (2) audit trails for tracking data manipulation and export procedures; (3) automated export procedures for seamless data downloads to common statistical packages; and (4) procedures for importing data from external sources [16].

Human research ethics boards at all involved institutions either approved the data collection and sharing protocol or deemed it exempt from review.

Reference ranges for abnormal values are provided in the results section, as the ranges varied between institutions.

## Results

### Case reports

We present findings from medical chart review of seven patients with NAGS deficiency, including two previously reported cases: case 3 [17] and case 6 [13]. Relevant family history was reported in three cases: consanguinity (case 2), a younger sibling who was diagnosed four years prior to proband’s diagnosis (case 5), and a cousin with seizures (case 6). In the other four cases, family history was negative for consanguinity, as well as for relevant presentations such as NAGS deficiency, hyperammonemia, and infant death. Suspected triggers for presenting episodes of hyperammonemia included: a vomiting illness that progressed to respiratory symptoms and lethargy (case 4), menstruation (case 5), and influenza B (case 6). There was no suspected trigger in the other four cases. Presenting symptoms and relevant abnormal laboratory findings are included in Table 1. Plasma glutamine concentration was elevated in all cases at presentation. Plasma citrulline concentration at presentation was in the normal range for three cases, elevated in one case, and only borderline decreased in two cases. Of note, mildly elevated urine orotic acid concentrations were found in three cases.

**Table 1:** Summary of case presentation and acute medical treatment

Case	Sex	Age at presentation	Presenting symptoms	Ammonia concentration	Other relevant abnormal test results at presentation	Treatment of presenting episode	Subsequent hyperammonemic episodes before treatment with carbamylglutatmate
1	Female	3 days	Apnea, convulsions, depressed consciousness, encephalopathy, lethargy, metabolic acidosis, oliguria, poor feeding/sucking, seizures, tachypnea, and unresponsiveness	Presenting: 1257 µmol/L (reference range < 77 µmol/L) Peak: 1500 µmol/L. During the presenting episode, hyperammonemia (> 100 µmol/L) was present for 6 days.	Glutamine (2132 µmol/L; ref: 246–984), urine orotic acid (14.8 mmol/mol creatinine; ref: 2.6–5.8)	Intravenous (IV) Sodium phenylbutyrate and sodium benzoate, hemodialysis followed by continuous veno-venous hemofiltration (CVVH), IV arginine, parenteral nutrition (TPN) with low protein and age-appropriate calories	None
2	Female	6 days	Abdominal distension, breathing difficulty, confusion, dehydration, drowsiness, encephalopathy, grunting, hypothermia, hypotonia, poor feeding, respiratory distress, and vomiting; Consanguinity	Presenting: >700 µmol/L (ref < 100 µmol/L) Peak concentration and number of days with elevated ammonia not available	Glutamine (793 µmol/L; ref: 229–684)	Hemodialysis for three days with two temporary discontinuations due to coagulated filters, IV sodium phenylacetate, and sodium benzoate.	Four additional episodes of hyperammonemia, with a total of three days with ammonia > 100 µmol/L
3^a^	Male	38 years	Asterixis, ataxia, behavioral problems, combativeness, confusion, depressed consciousness, drowsiness, encephalopathy, headaches, irritability, nausea, spasticity, and vomiting	Presenting: 434 µmol/L (ref: 15–50 µmol/L) at presentation. Peak concentration not available. During the presenting episode, hyperammonemia (> 100 µmol/L) was present for 8 days.	Glutamine (1062 µmol/L; ref: 104–750)	Sodium phenylbutyrate and citrulline (50 mg/kg/day)	Four additional episodes of hyperammonemia, but the concentration did not exceed 100 µmol/L
4	Male	25 months	Altered mental status, breathing difficulty, developmental delay, disconjugate gaze, encephalopathy, hyperpnea, irritability, metabolic acidosis, pallor, poor capillary refill, poor feeding, and vomiting	Presenting: 120 µmol/L (ref: 21–50 µmol/L) Peak: 185 µmol/L. During the presenting episode, hyperammonemia (> 100 µmol/L) was present for 2 days.	Glutamine (1448 µmol/L; ref: 410–700), citrulline (9 µmol/L; ref: 10–60), urine orotic acid (7.3 mmol/mol creatinine; ref: 0.7–5.1), urine ketones (3+), urine protein (1+)	Sodium phenylbutyrate, 10% dextrose water and ½ normal saline at 1.5 x maintenance (D10W ½ NS at 1.5 x M), and IV citrulline (170 mg/kg/day).	Four additional episodes of hyperammonemia, but the concentration did not exceed 100 µmol/L
5	Female	14 years	Abdominal pain, headaches, hypertonia, lethargy, nausea, protein aversion, and vomiting; family history of a sister diagnosed with NAGS deficiency as an infant.	Presenting: 95 µmol/L (ref: 11–35 µmol/L) Peak: 223 µmol/L. During the presenting episode, hyperammonemia (> 100 µmol/L) was present for 2 days.	Not available	Sodium phenylbutyrate and IV dextrose 12.5, ½ NS at two times maintenance	Several documented episodes but details are not available
6^b^	Female	10 years	Ataxia, encephalopathy, nausea, protein aversion, vomiting, dizziness, and syncope; family history of a cousin with seizures.	Presenting: 203 µmol/L (ref: 9–33 µmol/L) Peak: 293 µmol/L. During the presenting episode, hyperammonemia (> 100 µmol/L) was present for 2 days.	Glutamine (868 µmol/L, ref: 49–736), urine orotic acid (4.1 mmol/mol creatinine, ref: 0.2–1.2), ketones (4+)	Sodium benzoate, sodium phenylacetate, and IV arginine	18 additional episodes of hyperammonemia, with a total of 30 days with ammonia greater than 100 µmol/L
7	Male	2 years	Abdominal pain, confusion, feeding intolerance, poor feeding, seizures, and vomiting.	Details not available in chart	Glutamine (970 µmol/L; ref: 205–756), citrulline (63 µmol/L, ref: 12–55)	Sodium phenylbutyrate, sodium benzoate, and IV arginine	35 additional episodes of hyperammonemia, with a total of 19 days with ammonia > 100 µmol/L

*Notes* a. case previously reported [17]

b. case previously reported [13]

Initial treatments to reduce ammonia concentration are described in Table 1. Nutrition management was initiated prior to diagnosis of NAGS deficiency to reduce the risk of hyperammonemia (Table 2). Because diagnosis was a long process for many of the cases, management consisted of long-term protein restriction and medical management of episodes of hyperammonemia.

**Table 2:** Nutrition management

Case	Diet during initial hyper-ammonemic crisis	Pre-carbamylglutamate diet
1 Age at Dietary Record Calories (kcal/kg/day) Protein (g/kg/day) Arginine (mg/kg/day) Citrulline (mg/kg/day)	3 days 68 1 250 0	39 days 117 1.6 0 200
2 Age at Dietary Record Calories (kcal/kg/day) Protein (g/kg/day) Arginine (mg/kg/day) Citrulline (mg/kg/day)	6 days 120 0 0 0	24 days 130 1.0 250 0
3 Age at Dietary Record Calories (kcal/kg/day) Protein (g/kg/day) Arginine (mg/kg/day) Citrulline (mg/kg/day)	38 years 46 1.4 0 0	39 years Not available 1.0 0 50
4 Age at Dietary Record Calories (kcal/kg/day) Protein (g/kg/day) Arginine (mg/kg/day) Citrulline (mg/kg/day)	25 months 122 1.2 0 170	28 months 64 1.05–1.2 0 160
5 Age at Dietary Record Calories (kcal/kg/day) Protein (g/kg/day) Arginine (mg/kg/day) Citrulline (mg/kg/day)	14 years 35 0 0 150	18 years 30 0.5 0 150
6 Age at Dietary Record Calories (kcal/kg/day) Protein (g/kg/day) Arginine (mg/kg/day) Citrulline (mg/kg/day)	10 years 17 0 114 0	18 years 18.8 0.47 0 130
7 Age at Dietary Record Calories (kcal/kg/day) Protein (g/kg/day) Arginine (mg/kg/day) Citrulline (mg/kg/day)	2 years 28 0.36 0 210	22 years 28 0.45 0 200

Molecular diagnoses (Table 3) led to the initiation of carbamylglutamate in all seven cases. Between the presenting episode and initiation of carbamylglutamate, six patients had at least one additional episode of hyperammonemia (Table 3). The exception was case 1, who was hospitalized at day 3 with medical management of ammonia levels until carbamylglutamate was initiated after results of genetic testing.

**Table 3:** Summary of molecular testing, chronic medical treatment, and outcomes

Case	Molecular tests completed (days from initial sample collection to results)	Genotype	Time from presentation to carbamyl-glutamate initiation	Initial carbamyl-glutamate dose and method of administration	Most recent carbamyl-glutamate dose and method of administration	Current diet and supplements	Dietary plan for illness	Outcomes
1	Whole exome sequencing (34 days)	c1323C > G^a^ (pY441X) homozygous	36 days	Initial nasogastric (NG) tube dose of 188 mg/kg/day diluted in water	50 mg/kg/day divided into two doses per day mixed in applesauce	No protein restriction	Restrict protein and increase dose of carbamyl-glutamate	Seizure disorder, spastic cerebral palsy, and cortical visual impairment
2	*CPS1* gene testing and simultaneous *NAGS* gene testing (17 days)	c1228T > C^b^ (pS410P) homozygous	22 days	Initial NG tube dose of 100 mg/kg/day diluted in water, subsequently raised to 250 mg/kg/day	67 mg/kg/day divided into three doses per day, mixed into banana puree	No protein restriction 32 mg/kg/day Citrulline	No plan	None
3^a^	*OTC* gene testing, then *NAGS* gene testing (83 days)	IVS6 + 5G > A^c^ and c1298A > G^c^ (pE433G)	13 months	oral dose of 50 mg/kg/day diluted in water	50 mg/kg/day divided into three doses per day	1.3 g/kg/day protein (self-restricts)	No plan	None
4	*OTC* gene testing, then *NAGS* gene testing, then *CPS1* gene testing, then phenotypic core for *CPS1*, then *NAGS* (promoter), then whole genome sequencing (1011 days)	-3064 A > T^d^ and unspecified^e^	91 days	100 mg/kg/day Method not available	100 mg/kg/day Method not available	No protein restriction	No plan	None
5	Unknown	Not available	4 years	Not available	43 mg/kg/day divided into three doses per day	0.8 g/kg/day protein (self-restricts)	No nutrition plan. Increase dose of carbamyl-glutamate	Idiopathic adult-onset spastic diplegia
6^b^	*OTC* gene testing, then *NAGS* gene testing, then *CPS1* gene testing, then sequencing of *NAGS* gene regulatory region (3175 days)	-3063 C > A^f^ homozygous	8 years	Oral dose of 60 mg/kg/day	47 mg/kg/day Method not available	No protein restriction	No plan	Subglottic stenosis
7	OTC gene testing, then *NAGS* gene testing (865 days)	1312T > A^g^ (pY424N) Homozygous	20 years	Oral dose of 53 mg/kg/day diluted in water	57 mg/kg/day Method not available	No protein restriction	No plan	Diabetes and obesity

*Notes* a. This study

b. Mutation previously reported [7]

c. Case previously reported [17]

d. This study. Promoter mutation shown to abolish gene expression *in vitro*

e. This study. Variant in intron 1 shown to abolish gene expression in vitro. Mutation not specified in medical record

f. Case previously reported [13]

g. This study

After initiation of carbamylglutamate, only one case had additional documented episodes of hyperammonemia (case 1). This occurred at ages 7 and 12 months and was associated with interrupted access to carbamylglutamate. The first interruption was due to an insurance prior authorization and language barrier and the second interruption occurred when the child experienced increased seizures and sleepiness and the family was unable to administer carbamylglutamate. At the time of first admission to the Emergency Department, the patient’s ammonia concentration was 278 µmol/L, and the hyperammonemia resolved upon administration of carbamylglutamate. In the second instance, carbamylglutamate was initiated immediately on admission, prior to measurement of ammonia concentration. In addition, one patient (case 5) reported that she suspects episodes of mild elevations during periods of intercurrent illness.

The results of molecular testing, chronic medical management, and outcomes are presented in Table 3. Five cases liberalized dietary protein intake, while two continued to self-restrict protein intake. Citrulline supplementation was continued in one case. The lowest dose of carbamylglutamate given that was not associated with hyperammonemia was 43 mg/kg/day. After carbamylglutamate initiation, dietary changes were associated with weight loss and improved diabetes management in case 7.

### Biomarkers

Ammonia concentrations during the course of diagnosis and treatment are shown in Table 4. As indicated, ammonia concentrations decreased with the use of ammonia scavengers, although in three cases, ammonia concentration did not reach the normal range until after initiation of carbamylglutamate.

**Table 4:** Biomarker concentrations at presentation and during treatment, with reference ranges for abnormal values

Case	Biomarker	Presenting	Pre-carbamylglutamate	Post- carbamylglutamate	Most Recent
1	Plasma Glutamine (µmol/L) Plasma Arginine (µmol/L) Plasma Alanine (µmol/L) Plasma Citrulline (µmol/L) Urine Orotic Acid (mmol/mol creatinine) Plasma Ammonia (µmol/L)	2132 (246–984) Normal 1980 (124–573) Normal 14.8 (2.6–5.8) 1257 (< 77)	Normal Normal Normal 0 (6–52) 7.8 (2.6–5.8) 63 (Normal)	Normal Normal Normal Normal Normal 39 (Normal)	Normal Normal Normal Normal Normal 39 (Normal)
2	Plasma Glutamine (µmol/L) Plasma Arginine (µmol/L) Plasma Alanine (µmol/L) Plasma Citrulline (µmol/L) Urine Orotic Acid (mmol/mol creatinine) Plasma Ammonia (µmol/L)	793 (229–684) Normal 553 (128–447) 6 (6–35) Not done 700 (< 100)	Normal Normal 493 (128–447) Normal Not done 60 (Normal)	Normal 141 (14–111) 466 (128–447) Normal Not done 63 (Normal)	Normal Normal Normal Normal Not done 41 (Normal)
3	Plasma Glutamine (µmol/L) Plasma Arginine (µmol/L) Plasma Alanine (µmol/L) Plasma Citrulline (µmol/L) Urine Orotic Acid (mmol/mol creatinine) Plasma Ammonia (µmol/L)	1062 (104–750) Normal Normal Normal Normal 434 (15–50)	941 (104–750) Normal Normal Normal Normal 82 (15–50)	Normal Normal Normal Normal Normal 21 (Normal)	Normal Normal Normal Normal Normal 11 (Normal)
4	Plasma Glutamine (µmol/L) Plasma Arginine (µmol/L) Plasma Alanine (µmol/L) Plasma Citrulline (µmol/L) Urine Orotic Acid (mmol/mol creatinine) Plasma Ammonia (µmol/L)	1448 (410–700) Normal Normal 9 (10–60) 7.3 (0.7–5.1) 120 (21–50)	Normal Normal Normal 144 (10–60) Not done 43 (Normal)	Normal 190 (40–160) Normal 166 (10–60) Not done 22 (Normal)	400 (410–700) Normal Normal Normal Not done 25 (Normal)
5	Not available				
6	Plasma Glutamine (µmol/L) Plasma Arginine (µmol/L) Plasma Alanine (µmol/L) Plasma Citrulline (µmol/L) Urine Orotic Acid (mmol/mol creatinine) Plasma Ammonia (µmol/L)	868 (49–736) Normal Normal Normal 4.1 (0.2–1.2) 203 (9–33)	Normal Normal Normal Normal Not done 118 (9–33)	277 (414–863) Normal 208 (220–620) Normal Not done 7 (Normal)	Normal Normal Normal Normal Not done 44 (Normal)
7	Plasma Glutamine (µmol/L) Plasma Arginine (µmol/L) Plasma Alanine (µmol/L) Plasma Citrulline (µmol/L) Urine Orotic Acid (mmol/mol creatinine) Plasma Ammonia (µmol/L)	970 (205–756) Normal Normal 63 (12–55) Not done Not available	784 (205–756) Normal Normal 169 (12–55) Not done 54 (11–32)	Normal Normal Normal Normal Not done 9 (11–32)	Normal Normal Normal Normal Not done 32 (Normal)

Table 4 also includes concentrations for plasma glutamine, alanine, arginine, citrulline, and urine orotic acid. Treatment with carbamylglutamate resulted in normalization of these biomarkers. At the most recent measurement, all biomarkers were in the normal range, with the exception of case 4 for whom plasma glutamine was slightly below the normal range.

## Discussion

As the rarest urea cycle defect, with no common mutation and with variable symptom presentation, diagnosis and management of NAGS deficiency may be challenging. A review of 34 NAGS deficiency case reports is available, but was released over one decade ago [18]. In addition, reports that have combined data of patients with urea cycle disorders (UCD) in Europe [19] and in Japan [20] have not included patients with NAGS deficiency. Our systematic collection of patient data provides a cohesive picture of NAGS deficiency and will assist clinicians in refining best practices for treatment, especially as it relates to medication and nutritional management. Dietitians during the initial meeting all communicated lack of knowledge and confidence about guiding protein liberalization to patients upon initiation of carbamylglutamate. This case series addresses a continued critical knowledge gap in the description of NAGS deficiency and treatment.

In their review of 34 published cases of NAGS deficiency, Ah Mew and Caldovic made several conclusions [18] that are validated by our case series. First, they reported that the lowest reported daily dose of carbamylglutamate required to prevent hyperammonemia was 15 mg/kg/day. According to the manufacturer, the recommended daily maintenance dose of carbamylglutamate for patients with NAGS deficiency is 10 mg/kg to 100 mg/kg divided into two to four doses [21]. In our patient cases, doses ranged from 43 to 100 mg/kg/day and most were divided into two or three doses per day. In two cases, the carbamylglutamate was mixed with food (applesauce or banana puree). Although mixing with food is not recommended by the manufacturer, as the impact on efficacy has not been studied [21], mixing with food was part of successful treatment adherence for two of the cases we report. Second, Ah Mew and Caldovic reported that carbamylglutamate allowed for protein intake of 2 to 3 g/kg/day in some infant patients, although one patient became mildly ataxic after ingestion of more than 3.5 g/kg/day [18]. We found similar results. Of our seven cases, five have no protein restriction and two self-restrict at 0.6 and 1.3 g/kg/day. There were no reports among our cases of adverse events associated with high protein intake, intercurrent illness, or inadequate caloric intake. However, there were limited dietary data in the charts as monitoring by dietitians was generally limited after initiation of carbamylglutamate.

Third, Ah Mew and Caldovic concluded that protein restriction during illness may be prudent if poor oral tolerance prevents the administration of carbamylglutamate [18]. Two of our cases had formalized plans for illness (one reduced protein and both increased carbamylglutamate dose), but no cases had an established plan for lack of access to carbamylglutamate. Finally, the 2011 review reported that timely administration of carbamylglutamate to affected neonates who presented with acute hyperammonemia resulted in normal psychomotor development [18]. Among our cases, three had poor long-term medical outcomes, which do not appear to be related to hyperammonemia in two of the cases, but was due to a lapse in access to carbamylglutamate in the third (case 1).

Patient management and outcomes among our seven cases add support to the current guidelines recommending carbamylglutamate with nutrition management for controlling ammonia concentrations for patients with NAGS deficiency. None of our cases required other ammonia scavengers such as sodium benzoate and phenylbutyrate after the initiation of carbamylglutamate. Likewise, six of the seven cases were able to discontinue arginine and citrulline supplements, although one continued to use citrulline supplements as part of disease management. Therefore, this report endorses the guidelines recommending carbamylglutamate as an effective and well-tolerated treatment option for individuals with NAGS deficiency.

While there were delays in initiation of carbamylglutamate in several of our cases until after diagnosis of NAGS deficiency, current guidelines recommend the administration of carbamylglutamate in all cases of unexplained hyperammonemia (> 100 µmol/L), with long-term continuation of carbamylglutamate if NAGS deficiency is confirmed [9]. This suggests that widespread dissemination of guidelines for the use of carbamylglutamate in cases of unexplained hyperammonemia is warranted.

Carbamylglutamate has also been used in the treatment of other disorders, most notably organic acidurias. In 2021, the US Food and Drug Administration approved carbamylglutamate for the treatment of acute hyperammonemia associated with propionic acidemia (PA) and methylmalonic acidemia (MMA) [22]. The mechanism for the effectiveness of carbamylglutamate in these conditions is still not clear. It has been proposed that accumulation of propionyl-CoA or methylmalonyl-CoA, respectively, causes competitive inhibition of NAGS [23], which may be compensated for by carbamylglutamate.

This case series includes three previously unreported mutations. There was no standardized approach to genetic testing for the cases in this report. A definitive diagnosis of NAGS deficiency, based on DNA, was a rate-limiting factor, often taking years, with single gene tests ordered sequentially. Gene panels for hyperammonemia or whole exome sequencing may address this issue, but may miss some mutations if regulatory regions are not included. Therefore, whole genome sequencing may be considered an efficient option. Additional data on the genetic diagnosis process, including turnaround times, will be important and may help to inform evidence-based guidelines on DNA testing for patients presenting with acute hyperammonemia. Current guidelines for urea cycle disorders include DNA testing for confirmation of NAGS deficiency, although the specific methodology for the genetic testing is not outlined [9].

Ah Mew and Caldovic describe the biochemical presentation of NAGS deficiency as including elevated plasma ammonia and glutamine concentrations, and low-to-normal concentrations of other urea cycle intermediates, while plasma citrulline is frequently low or undetectable [18]. Plasma glutamine was elevated in all of our cases at presentation, as expected. However, citrulline was normal at presentation in three of our cases, borderline in two, and high in one. In one case (case 1), citrulline was reduced from normal to zero after hemodialysis without citrulline supplementation and prior to initiation of carbamylglutamate. Furthermore, while it has been commonly accepted that NAGS deficiency presents with low or normal urine orotic acid prior to administration of carbamylglutamate [9], we report slightly elevated urine orotic acid concentration in three of the seven cases. Marginal to mild elevations have also been previously reported in three other patients with NAGS deficiency [15, 24]. The cause and any clinical consequence of this elevation remain to be understood, but our results indicate that mild elevations in urine orotic acid concentration should not be used to rule out NAGS deficiency.

## Conclusions

In conclusion, we report on the diagnosis and management history of seven cases of NAGS deficiency in the US and Canada. By incorporating an in-person meeting with case presentations and discussion into our methodology, the providers learned from each other and identified topics for comprehensive chart review and data collection strategies. The biochemical profiles during presentation in some of our cases did not align with previously published NAGS deficiency cases, suggesting further case review and consensus is needed to fully characterize biomarkers suggestive of NAGS deficiency. Despite the availability and efficacy of carbamylglutamate for the treatment of NAGS deficiency, there remains heterogeneity in management approaches, with differences in protein intake and micronutrient supplementation. In addition to developing formalized plans for disease management during times of illness, which may include protein restriction and increased doses of carbamylglutamate, we also recommend that clinicians work with families to develop nutrition management plans for unexpected lapses in access to carbamylglutamate. In addition, while protein restriction is generally not needed when carbamylglutamate is used during chronic management, the upper limit of protein and caloric intake needs to be further assessed with ongoing dietary management to understand the potential impact of higher levels of protein intake. NAGS deficiency is the only urea cycle disorder which can be specifically and effectively treated with medication, with the potential of having an unrestricted diet. Early and continuous treatment with carbamylglutamate optimizes outcome. However, there remains a need for further dissemination of and education related to the guidelines for the use of carbamylglutamate during unexplained hyperammonemia.

## Data Availability

The datasets generated and analyzed during the current study are not publicly available because they were created under data sharing agreements between the partnering institutions which preclude the sharing of data to additional parties. Data are however available from the authors upon reasonable request and with permission of individual institutions.

## Abbreviations

ALT: Alanine transaminase
AST: Aspartate aminotransferase
BUN: Blood urea nitrogen
CPS1: Carbamoyl phosphatase synthetase 1
CVVH: Continuous veno-venous hemofiltration
FDA: Food and Drug Administration
HIPAA: Health Insurance Portability and Accountability Act
IMD: Inherited metabolic disorder
IV: Intravenous
MMA: Methylmalonic acidemia
NAG: N-acetylglutamate
NAGS: N-acetylglutamate synthase
PA: propionic acidemia
TPN: Total parenteral nutrition
TSH: Thyroid stimulating hormone
US: United States
